# Magnet-Bead Based MicroRNA Delivery System to Modify CD133^+^ Stem Cells

**DOI:** 10.1155/2016/7152761

**Published:** 2016-10-04

**Authors:** Paula Müller, Natalia Voronina, Frauke Hausburg, Cornelia A. Lux, Frank Wiekhorst, Gustav Steinhoff, Robert David

**Affiliations:** ^1^Reference and Translation Center for Cardiac Stem Cell Therapy (RTC), Department of Cardiac Surgery, Rostock University Medical Center, Schillingallee 69, 18057 Rostock, Germany; ^2^Physikalisch-Technische Bundesanstalt, Abbestraße 2-12, Berlin 10587, Germany

## Abstract

*Aim*. CD133^+^ stem cells bear huge potential for regenerative medicine. However, low retention in the injured tissue and massive cell death reduce beneficial effects. In order to address these issues, we intended to develop a nonviral system for appropriate cell engineering.* Materials and Methods*. Modification of human CD133^+^ stem cells with magnetic polyplexes carrying microRNA was studied in terms of efficiency, safety, and targeting potential.* Results*. High microRNA uptake rates (~80–90%) were achieved without affecting CD133^+^ stem cell properties. Modified cells can be magnetically guided.* Conclusion*. We developed a safe and efficient protocol for CD133^+^ stem cell modification. Our work may become a basis to improve stem cell therapeutical effects as well as their monitoring with magnetic resonance imaging.

## 1. Introduction

The transplantation of stem cells for the recovery of damaged tissue represents a promising strategy in regenerative medicine. Adult stem cells bear considerable potential since they are easily accessible from patients or healthy donors without posing ethical conflicts [1]. Among these, cells expressing the highly conserved transmembrane CD133 antigen (prominin-1) represent a heterogeneous stem and progenitor cell population capable of differentiating into haematopoietic, endothelial, and myogenic lineages [2–4]. We and others have shown that their regeneration potential is mainly based on cytoprotective effects and their contribution to neovascularization processes through differentiation into newly forming vessels and activation of proangiogenic signaling by paracrine mechanisms [5–8]. The high therapeutic relevance of these cells is reflected in more than 30 approved clinical trials (ClinicalTrials.gov) using adult CD133^+^ stem cells for the treatment of various degenerative diseases. For instance, remarkably promising results have been reported for the treatment of chronic ischemic cardiomyopathy. An increase of left ventricular ejection fraction (LVEF) by 5-6% following intramyocardial application led to the approval of first phase III clinical studies in the field of CD133 research [9–13]. However, despite the rapid translation from bench to bedside, broad clinical application of stem cells is still hampered by low retention in the organ of interest and massive initial cell death [6, 14, 15]. Engineering of cells prior to transplantation can address all of the mentioned challenges. To accomplish this objective, the development of a safe and efficient way of CD133 modification for relevant improvement of their properties is required.

The retention of therapeutic cells at the site of interest is a fundamental prerequisite for an effective therapy [16]. Nevertheless, in highly perfused organs, such as the brain and the heart, 90–99% of transplanted cells are typically lost during the first 1-2 h, independently of the cell type and application route [17–27]. To overcome this immense washout, magnetic targeting of iron oxide labeled stem cells can be applied as an innovative noninvasive strategy to enhance immediate retention and therefore improve long-term engraftment and functional benefits [28–32]. In addition, cells which are magnetically labeled can be tracked and monitored using magnetic resonance imaging (MRI) [33] or the novel imaging modality magnetic particle imaging (MPI) [34, 35].

Another major limitation of stem cell therapy is the massive cell death after transplantation due to the hostile, inflammatory environment of the target tissue [36]. Several strategies have been developed to improve the survival of grafted cells, including their preconditioning by hypoxia, heat shock, or cytokine treatment [37, 38]. In addition, it has been successfully demonstrated in various studies that antiapoptotic microRNAs (miRs) both inhibit apoptosis* in vitro* and increase cell engraftment following transplantation* in vivo* [39]. MiRs have been proven to be important translational controllers of stem cell fate and behaviour, avoiding the hazard of stable integration into the genome [40]. Therefore, these small molecules are ideal candidates for cell engineering aiming at survival improvement.

Currently, delivery of nucleic acids into hard to transfect primary stem cells is almost exclusively based on recombinant viruses which are the most efficient vehicles so far [14, 41]. However, uncontrollable gene expression, pathogenicity, immunogenicity, and insertional mutagenesis of viral vectors remain major obstacles for widespread clinical translation [41, 42]. Consequently, the necessity of safer gene delivery methods has led to the development of various nonviral systems which are nonpathogenic, nonimmunogenic, and not limited by the size of delivered genetic material [43].

**Figure 1:**
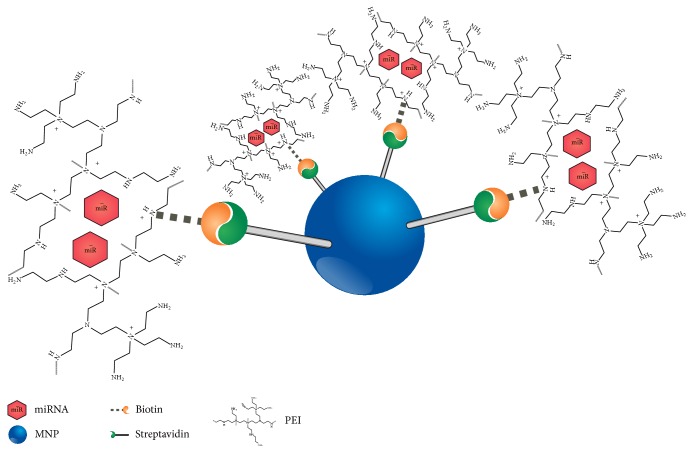
Schematic structure of superparamagnetic transfection complexes. Complexes are composed of a streptavidin-coated magnetic iron oxide nanoparticle (MNP) and biotinylated polyethylenimine (PEI), which condenses miR through electrostatic interactions.

Nowadays, polyethylenimine (PEI) is one of the most efficient polymers for miR delivery, promoting nucleic acid protection against degradation, cellular uptake, and intracellular release [44]. The implementation of miR-PEI constructions in first clinical trials is demonstrating their high biocompatibility [45]. In our group, a vector has been designed, which consists of biotinylated PEI bound to streptavidin-coated iron oxide magnetic nanoparticles (MNPs) (Figure 1). During previously carried trials, the group worked on the adjustment of vector efficiency and safety. During these studies, it has been demonstrated that pDNA and miR can be efficiently delivered and processed in human mesenchymal stem cells (hMSCs) [46, 47].

In this study, we worked on the development of an efficient strategy for magnet-bead based miR delivery into highly clinically relevant cell type, CD133^+^ stem cells. First, we have demonstrated that optimized transfection complexes are suitable for sufficient miR delivery into bone marrow (BM) derived CD133^+^ stem cells without affecting stem cell marker expression and haematopoietic differentiation capacity. Moreover, we showed that modified cells can be magnetically guided* in vitro*. These two achievements together form an essential prerequisite for further preclinical testing.

## 2. Materials and Methods

### 2.1. BM Specimens

BM was obtained from informed donors who gave their written consent to use their samples for research according to the Declaration of Helsinki. The study was approved by the ethical committee of the University of Rostock in 2010 and renewed in 2015 (registration number A 2010 23). Sternal BM aspirates were obtained from patients undergoing coronary artery bypass grafting at the Department of Cardiac Surgery (University Hospital Rostock, Germany). To prevent coagulation, heparin sodium (250 i.E./mL BM) (Ratiopharm GmbH, Germany) was used.

### 2.2. CD133^+^ Cell Isolation

Mononuclear cells (MNCs) were isolated by layering patient derived BM on Pancoll human separation solution (Pan Biotech GmbH, Germany) and subsequent density gradient centrifugation. CD133^+^ cells were magnetically enriched using the MACS CD133 MicroBead Kit (Miltenyi Biotec GmbH, Germany) following the manufacturer's instructions. For further experiments, only CD133^+^ cell fractions with viability and purity (expression of stem cell surface markers) higher than 80% were used.

### 2.3. Assay to Address Viability and Expression of Stem Cell Surface Markers of CD133^+^ Cells

Cell viability and stem cell surface marker expression were analysed by flow cytometry 0 h, 18 h, and 24 h after isolation. For staining, samples were treated with the following antibodies: anti-CD34-FITC (clone: AC136), anti-CD133/2-PE (clone: 293C3), isotype control mouse IgG 2b-PE (Miltenyi Biotec GmbH), anti-CD45-APC-H7 (clone: 2D1), and 7-AAD (BD Biosciences, Germany). To reduce unspecific bindings, FcR blocking reagent (Miltenyi Biotec GmbH) was added. After incubation for 10 min at 4°C, samples were measured with LSR-II flow cytometer (BD Biosciences) and data analysis was realised with FACSDiva software (BD Bioscience). For evaluation, Boolean gating strategy was arranged on the ISHAGE guidelines for CD34^+^ cell analysis [48] in following order:

1. Step  1: selection of cell population (Figure S1A, in Supplementary Material available online at http://dx.doi.org/10.1155/2016/7152761).
2. Step  2: selection of CD45^+^ cells (Figure S1B).
3. Step  3: selection of viable CD45^+^ cells (Figure S1C).
4. Step  4: selection of viable CD45^+^/CD34^+^ cells (Figure S1D).
5. Step  5: selection of viable CD45^+^/CD34^+^/CD133^+^ cells (Figure S1E).

Calculation of cell viability and surface marker integrity was based on the following equations:

(1) Viability%=viable  CD45+  cellsCD45+  cells×100,Surface  marker  pattern%=Viable  CD45+/CD34+/CD133+  cellsViable  CD45+  cells×100.

### 2.4. Formation of Polyplexes and Transfection

During transfection, Cy3™ dye-labeled Pre-miR Negative Control #1 (Ambion, USA) was delivered to examine uptake efficiency, cytotoxicity, and magnetic targeting. Pre-miR™ miRNA Precursor Molecules Negative Control #1 (Ambion, USA) was used for flow cytometry gating controls, colony-forming unit (CFU) assays, surface marker expression analysis, and intracellular visualization of complexes. Both miRs are double-stranded RNA molecules designed to mimic endogenous mature miRNAs.

In order to ensure effective binding to streptavidin-coated MNPs, branched PEI with a molecular weight of 25 kDA (Sigma-Aldrich, USA) was biotinylated using EZ-Link Sulfo-NHS-LC-Biotin (Thermo Fisher Scientific GmbH, Germany) according to the manufacturer's instructions and previous reports [49]. Minor changes were made in solvents (water instead of DMSO) and purification method: size-exclusion chromatography with PD-10 Desalting columns (GE Healthcare, UK) was used instead of dialysis as described elsewhere [50]. Final concentration of amine groups was measured by ninhydrin assay (2% ninhydrin reagent, Sigma-Aldrich) and the biotinylation degree was determined as 1.585 ± 0.018 mmol biotin/mmol PEI in HABA assay using Pierce Biotin Quantitation Kit (Thermo Scientific).

Streptavidin MagneSphere® Paramagnetic Particles (Promega Corporation, USA) were filtered through 0.45 *μ*m Millex-HV PVDF Syringe Filter Units (EMD Millipore Corporation, USA) to remove bigger aggregates and particles. Biotinylated PEI and filtered MNPs were stored at 4°C until usage.

For the formation of polyplexes, miR and PEI were diluted in equal volumes of 5% glucose solution (MP Biomedicals, Germany) in appropriate amounts to prepare different molar ratios of PEI nitrogen and miR phosphate (N/P ratios). After vigorous mixing, miR/PEI solution was incubated for 30 min at RT.

In order to form magnetic polyplexes, MNPs were incubated in an ultrasonic bath for 20 min every time before use to remove formed clusters. Prepared this way, MNPs were mixed with previously prepared miR/PEI complexes in different concentrations and the solution was incubated for 30 min at RT.

Freshly isolated 5 × 10^4^ CD133^+^ cells were seeded for transfection in a 24-well plate and prepared miR/PEI or miR/PEI/MNP complexes were added immediately. After incubation for 18 h at 37°C and 5% CO_2_ in Dulbecco's Modified Eagle Medium (DMEM, Pan Biotech GmbH) supplemented with 1% penicillin/streptomycin (PAA Laboratories GmbH, Germany) and 2% fetal bovine serum (FBS, Pan Biotech GmbH), cells were directly used for measurements or fresh medium was supplied. In order to further optimize culture conditions for CD133^+^ cells, in one set of tests, StemSpan™ H3000 culture medium (STEMCELL Technologies Inc., Canada) supplemented with StemSpan CC100 (STEMCELL Technologies Inc.) was used for incubation (see Figure 8).

### 2.5. Uptake Efficiency and Cytotoxicity of Transfection Complexes

For the quantification of uptake efficiency and cytotoxicity of different transfection complex formulations, CD133^+^ cells were stained 18 h after transfection for 10 min at 4°C with LIVE/DEAD® Fixable Near-IR Dead Cell Stain Kit (Molecular Probes, USA) and fixed with 4% formaldehyde solution (FA) (Merck Schuchardt OHG, Germany). Samples were measured with LSR-II flow cytometer and data were analysed with FACSDiva software. The representative gating strategy is depicted in Figure S2.

Qualitative analysis of transfected CD133^+^ cells was carried out 18 h after transfection based on Cy3-labeled miR. For this purpose, cells were washed once with 2% FBS in phosphate buffered saline (PBS, Pan Biotech GmbH) in order to remove noninternalized particles and fixed with 4% FA for 20 min. Afterwards, cells were spun down to a coverslip and washed again with PBS. Then, the coverslip was mounted with Fluoroshield™ containing DAPI (Sigma-Aldrich) on microscope slides. These prepared samples were subjected to laser scanning confocal microscopy (40x oil immersion) in the tile-scan mode in order to acquire larger areas of 1062.33 *μ*m × 1062.33 *μ*m. In addition, in order to ensure that exclusively internalized Cy3 signal was analysed, *z*-stacks were recorded of approximately 7 *μ*m depth.

### 2.6. Magnetic Targeting

The verification of magnetic cell targeting was performed with CD133^+^ cells transfected at optimized conditions (20 pmol miR; N/P ratio 7.5; 3 and 5 *μ*g/mL MNPs). 18 h after transfection, cells were transferred to a 12-well plate and a magnetic field was applied locally using magnetic plate (OZ Biosciences, France; field strengths 70–250 mT and 50–130 T/m, resp. [51]). 24 h after incubation at 37°C and 5% CO_2_, cell numbers in the area with (+*M*) and without (−*M*) magnet were counted by microscopic observation using the LSM 780 ELYRA PS.1 system and image files were analysed with ZEN 2011 software (Carl Zeiss Microscopy GmbH, Germany) and ImageJ 1.48 (NIH, USA). Magnetic targeting ratios were calculated by dividing obtained cell numbers of both areas (+*M*/−*M*).

### 2.7. Magnetic Particle Spectroscopy to Define Magnetic Loading of CD133^+^ Cells

Cell samples modified under the conditions defined as optimal for transfection and magnetic cell guidance were investigated by magnetic particle spectroscopy (MPS) to quantify the magnetic iron loading of CD133^+^ cells with miR/PEI/MNP complexes. MPS is a sensitive magnetic detection method that allows for the specific quantification of the magnetic nanoparticle iron content without being affected by cells or suspension medium [52, 53]. After incubation of 18 h, untransfected (control) and transfected CD133^+^ cells were collected and washed with 2% FBS in PBS. Subsequently, cell samples of about 5 × 10^5^ cells were resuspended in PBS, fixed with 4% FA, and transferred into 0.2 mL tubes for the measurements by a commercial MPS device (Bruker Biospin, Germany) operating at a magnetic field *B*_drive_ = 25 mT and a frequency *f*_0_ = 25 kHz. This device determines the nonlinear dynamic magnetic moments of the sample at higher (odd) harmonics of *f*_0_. The iron quantification of the samples is carried out by the third harmonic *A*_3_ of the MPS spectrum which is normalized to the corresponding *A*_3,ref_ of the MNP reference sample of known iron amount (3.2 *μ*g for MNPs only reference and 2.5 *μ*g for the CD133 MicroBeads reference) as described before [54]. The ratio of the fifth to third harmonic *A*_5_/*A*_3_ is used as a characteristic fingerprint to identify the magnetic nanoparticle type in the sample.

### 2.8. Intracellular Visualization of Transfection Complexes

For visualization of complexes, Pre-miR miRNA Precursor Molecules Negative Control #1 (Ambion) was labeled with Cy5™ dye using Label IT^®^ miRNA Labeling Kit (Mirus Bio LLC, USA) as recommended in the manufacturer's instructions. Redundant dye was removed using supplied purification column. The Cy5-labeled miR final concentration was measured spectrophotometrically in NanoDrop ND-1000 (Thermo Scientific, USA) and samples were stored at −20°C protected from light until further use.

PEI was stained using FluoReporter® Oregon Green® 488 Protein Labeling Kit (Molecular Probes) according to the manufacturer's protocol. Therefore, PEI was mixed with 1 M sodium bicarbonate solution and incubated for 1 h with Oregon Green stock solution (10 mg/mL in DMSO). Unreacted dye was removed by size-exclusion chromatography using PD-10 Desalting columns (GE Healthcare, UK). 488-labeled PEI concentration was defined in the ninhydrin assay and aliquots were further stored at 4°C protected from light.

MNPs were stained using Atto 565 dye conjugated to biotin (ATTO-TEC GmbH, Siegen, Germany). For this purpose, Atto 565 dye and MNPs (ratio of 1 : 1000 w/w) were mixed simultaneously with prepared miR/PEI complexes and incubated for 30 min in the dark.

To monitor the intracellular distribution of complexes, 5 × 10^4^ CD133^+^ cells were transfected with fluorescently labeled complexes at previously optimized conditions (20 pmol miR; N/P ratio 7.5; 3 *μ*g/mL MNPs). 18 h after transfection, samples for microscopy were prepared as described above.

First, in order to assess the intracellular localisation of all delivery vector parts, confocal laser scanning microscopy (LSM) was performed using ELYRA PS.1 LSM 780 system. ZEN software (Carl Zeiss Microscopy GmbH) was applied for image processing.

Further, the localisation of miR/PEI/MNP was studied more in detail using 3-dimensional structured illumination microscopy (SIM). Four-colour imaging was performed using 100x alpha Plan-Apochromat 1.46 objective (oil immersion): 405, 488, 561, and 633 nm laser lines for excitation. SI raw data was acquired in *z*-stacks with a 16-bit depth at 3 angles, 3 phases, with averaging 4; 23 *μ*m grid was applied for 405 laser line, 34 *μ*m—488, 42 *μ*m—561, and 51 *μ*m—633. These SI raw datasets were computationally reconstructed by ZEN software (Carl Zeiss Microscopy GmbH). Presented final images were obtained as a result of alignment of maximum projections created separately for each channel.

### 2.9. Haematopoietic CFU (CFU-H) Assay

In order to monitor haematopoietic differentiation capacity of miR-modified CD133^+^ cells, CFU-H assays were performed. 18 h after transfection with Pre-miR miRNA Precursor Molecules Negative Control  #1, cells were mixed with MethoCult H4434 Classic (STEMCELL Technologies) and 1 × 10^3^ cells were seeded per 35 mm dish. Formed colonies were identified and counted for the assessment after 14 d incubation at 37°C and 5% CO_2_. As recommended by manufacturer, all samples were examined in duplicate.

### 2.10. Statistical Analysis

Statistical analysis was performed using Student's *t*-test with SigmaPlot 11.0 (Systat Software GmbH, Germany). All values are presented as mean ± standard error of the mean (SEM). Values with *p* ≤ 0.05 (*∗*; #), *p* ≤ 0.01 (*∗∗*; ##), and *p* ≤ 0.001 (∗∗∗; ###) were considered to be statistically significant. For every experiment, different BM donors (*n*) were used.

## 3. Results

### 3.1. Optimization of miR/PEI Complexes for Transfection

**Figure 2:**
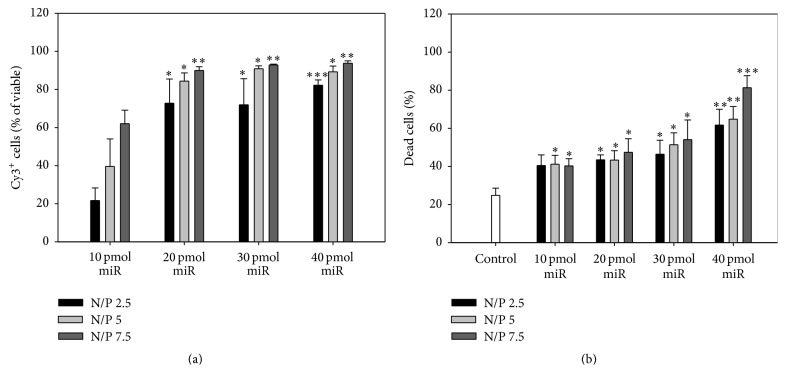
Optimization of CD133^+^ transfection with different compositions of miR/PEI complexes. Cells were transfected with Cy3-labeled complexes consisting of four different miR amounts (10, 20, 30, and 40 pmol) and three different N/P ratios (2.5, 5, and 7.5). Uptake efficiency (a) and cytotoxicity (b) were measured 18 h after transfection by flow cytometry. Untransfected cells were used as control. Values are presented as mean ± SEM; *n* = 4; statistic was performed versus 10 pmol miR with respective N/P ratio (a) and versus control (b); ^*∗*^ *p* ≤ 0.05; ^*∗∗*^ *p* ≤ 0.01; ^*∗∗∗*^ *p* ≤ 0.001.

In order to optimize the transfection of CD133^+^ cells, different compositions of miR/PEI complexes consisting of four different miR amounts (10, 20, 30, and 40 pmol per 5 × 10^4^ cells) and three different N/P ratios (2.5, 5, and 7.5) were tested in terms of uptake efficiency and cytotoxicity (Figure 2). In all measurements, untransfected cells served as internal control representing the cytotoxic effect of the isolation and cultivation procedure.

Complexes with the smallest miR amount (10 pmol) showed the lowest uptake rates (ranging between ~20 and 60% Cy3^+^ cells) and a minor increased cytotoxicity (~40% dead cells) compared to the control (~25% dead cells). As expected, complexes consisting of higher miR amounts led to a significantly increased uptake (up to ~95% Cy3^+^ cells, 40 pmol miR; N/P 7.5) but also resulted in increasing cytotoxic effects (up to ~80% dead cells, 40 pmol miR; N/P 7.5) because higher PEI amounts were required. Therefore, complexes composed of 20 pmol miR were considered as optimal for transfection of CD133^+^ cells representing a balance between increase in uptake rates (~75–90% Cy3^+^ cells) and compromised cell survival.

### 3.2. MiR/PEI/MNP Complexes Are Suitable for CD133^+^ Stem Cell Transfection

**Figure 3:**
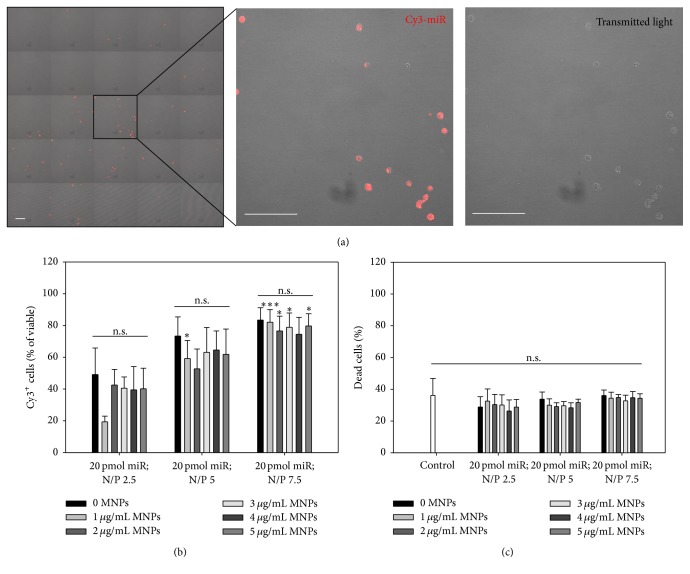
Optimization of CD133^+^ transfection using different compositions of miR/PEI/MNP complexes. Cells were transfected with Cy3-labeled complexes consisting of 20 pmol miR, three different N/P ratios (2.5, 5, and 7.5), and six different MNP amounts (0, 1, 2, 3, 4, and 5 *μ*g/mL). Representative images confirming intracellular localisation of complexes were taken 18 h after transfection using laser scanning confocal microscopy (a). Uptake efficiency (b) and cytotoxicity (c) were measured 18 h after transfection by flow cytometry. Untransfected cells were used as control. Values are presented as mean ± SEM; *n* = 4; statistic was performed versus 20 pmol miR, N/P ratio 2.5 with respective MNP amount (indicated as *∗*) or within the same N/P ratio (a) and versus control (b); ^*∗*^ *p* ≤ 0.05; ^*∗∗*^ *p* ≤ 0.01; ^*∗∗∗*^ *p* ≤ 0.001. Scale bar = 50 *μ*m.

To achieve the possibility of magnetic targeting, previously selected polyplexes (20 pmol miR; N/P ratio 2.5, 5, and 7.5) were complemented by MNPs in six different concentrations (0, 1, 2, 3, 4, and 5 *μ*g per mL of prepared miR/PEI mixture). The influence of MNPs with respect to uptake efficiency and cytotoxicity was analysed by flow cytometry and confocal microscopy (Figure 3). Again, untransfected cells served as internal control.

Uptake rates of miR/PEI/MNP complexes did not significantly differ from respective miR/PEI complexes (0 MNPs), whereas raising N/P ratios (2.5, 5, and 7.5) led to increasing uptake efficiencies (~40%, ~60%, and ~80% Cy3^+^ cells, resp.) (Figure 3(b)). Representative images illustrate the intracellular localisation of labeled miR-Cy3 and its high uptake rates when a complex formulation of 20 pmol miR N/P ratio 7.5 combined with 3 *μ*g/mL MNPs was applied (Figure 3(a)). Furthermore, no significant changes in cytotoxicity were determined between the control (36 ± 11% dead cells) and transfected cells (ranging between ~25 and 35% dead cells) (Figure 3(c)). In following experiments, magnetic complexes composed of 20 pmol miR and N/P ratios 5 and 7.5 combined with 3 and 5 *μ*g/mL MNPs were used with respect to their highest uptake rates and low cytotoxicity compared to the control.

### 3.3. MiR/PEI/MNP-Modified CD133^+^ Stem Cells Can Be Efficiently Guided via a Magnetic Field

**Figure 4:**
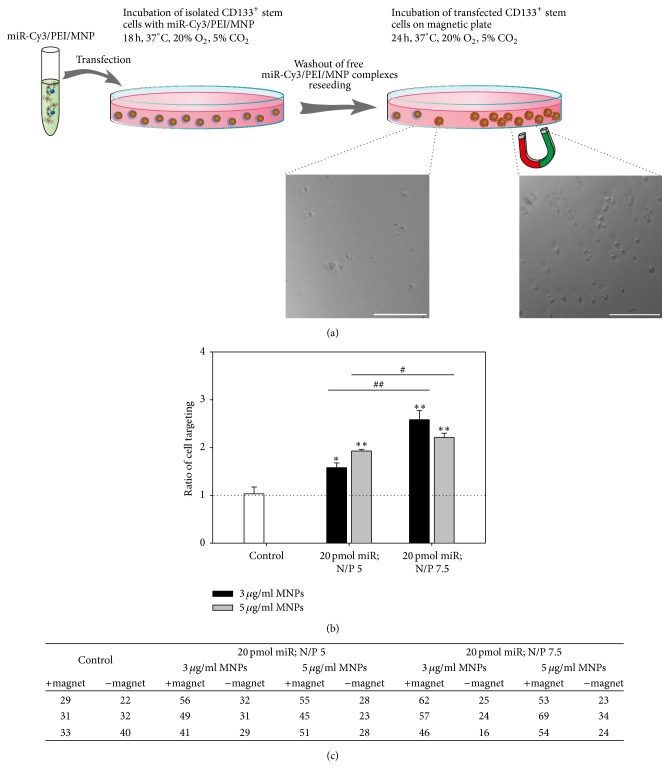
Magnetic targeting of modified CD133^+^ cells. Cells were transfected with Cy3-labeled complexes consisting of 20 pmol miR, two different N/P ratios (5 and 7.5), and MNP amounts (3 and 5 *μ*g/mL). 18 h after transfection, a local magnetic field was applied for 24 h. Pictures were taken from areas with and without magnet (a). Cell numbers in both areas were counted (c) and magnetic targeting ratios were calculated (b). Untransfected cells were used as control. Values are presented as mean ± SEM; *n* = 3; statistic was performed versus control (indicated as ∗) or within respective MNP amounts (indicated as #); ^*∗*; #^ *p* ≤ 0.05; ^*∗∗*; ##^ *p* ≤ 0.01; ^*∗∗∗*; ###^ *p* ≤ 0.001. Scale bar = 100 *μ*m.

To investigate the influence of a magnetic field on the guidance of cells transfected with optimized miR/PEI/MNP complexes, a magnet was applied locally under the well plate for 24 h (Figure 4(a)).

In case of untransfected cells, almost the same cell numbers were detected in both areas (with and without magnet) (Figure 4(c)). These results indicate that MACS CD133 MicroBeads alone are not sufficient for magnetic targeting of cells. In contrast, cells transfected with magnetic complexes composed of 20 pmol miR and N/P ratios 5 and 7.5 combined with 3 and 5 *μ*g/mL MNPs showed higher cell numbers in the area of the magnet (+ magnet) than in the area without magnet (− magnet). Correspondingly, transfected cells showed significantly higher magnetic targeting ratios (ratios between 1.6 and 2.6) than untransfected cells (ratio of 1 ± 0.12) (Figure 4(b)). Moreover, transfection complexes with N/P ratio 7.5 led to significantly higher targeting ratios (2.6 ± 0.17 and 2.2 ± 0.08 with 3 and 5 *μ*g/mL MNPs) than complexes with N/P ratio 5 (1.6 ± 0.09 and 1.9 ± 0.02 with 3 and 5 *μ*g/mL MNPs). Therefore, complexes composed of 20 pmol miR and N/P ratio 7.5 combined with 3 or 5 *μ*g/mL MNPs are assumed to be most suitable for targeting of transfected cells in a magnetic field and were used for further experiments.

### 3.4. Intracellular Iron Concentration

For the conditions defined as optimal for transfection and magnetic cell guidance, the amount of intracellular iron has been quantified using magnetic particle spectroscopy (MPS). As a result, we observed that a cellular uptake of 0.155 ± 0.0419 pg iron per cell could be achieved with our protocol for cells transfected by miR/PEI/MNP complexes. Importantly, the corresponding *A*_5_/*A*_3_ values in the range 20.5 ± 3.1% allowed for a unique identification of successfully uptaken miR/PEI/MNP complexes, avoiding false positive signals emanating from magnetic cell isolation beads. MPS allows this discrimination due to the fact that untransfected as well as transfected cell samples yield signals with *A*_5_/*A*_3_ values in the range of 27 to 30% which can be attributed exclusively to CD133 MicroBeads used for cell isolation.

### 3.5. Intracellular Visualization of Transfection Complexes

For intracellular detection of the optimized transfection complexes, CD133^+^ stem cells were transfected with labeled miR/PEI/MNP complexes. The analysis of cellular complex uptake 18 h after transfection with confocal microscopy allowed for concluding that all vector parts are sufficiently delivered into cells (Figure S3). This additionally validates the flow cytometry results: when proper washing steps are applied, complexes from the surface are removed, whereas intracellular uptake is being analysed.

**Figure 5:**
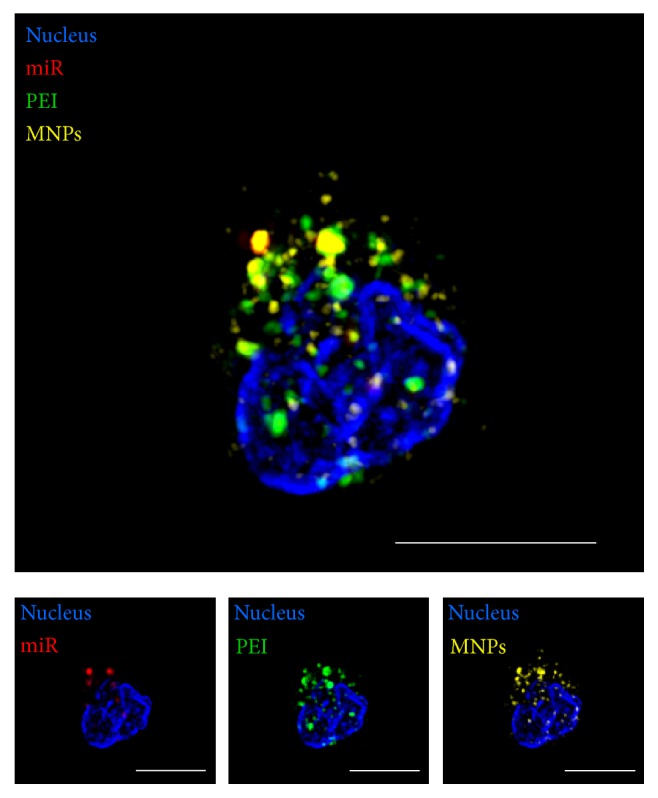
Intracellular visualization of transfection complexes. Cells were transfected with optimized fluorescently labeled miR/PEI/MNP (20 pmol miR; N/P ratio 7.5; 3 *μ*g/mL MNPs) complexes. miR staining was performed with Cy5 dye (red). PEI was labeled Oregon Green 488 (green). MNPs were stained with Atto 565 (yellow). Nuclei were counterstained with DAPI (blue). Representative images were taken 18 h after transfection using structured illumination microscopy (SIM). Scale bar = 5 *μ*m.

In order to monitor vector distribution with higher resolution, SIM was performed (lateral resolution up to 100 nm compared to 200–500 nm for confocal). As a result, we observed that 18 h after transfection miR/PEI/MNP complexes were mainly detected in the cytoplasm. All vector parts were located in the perinuclear region with no discernible differences between them (Figure 5). The signal of PEI was always colocalised with MNP and miR. Formed structures 100–400 nm in size were located in the other focal plane compared to nucleus as recording of *z*-stacks demonstrated.

### 3.6. miR/PEI/MNP Complexes Have No Negative Influence on Cell Viability and Surface Marker Pattern

To evaluate the impact of transfection and subsequent 18 h culturing time on cell viability and surface marker pattern, flow cytometry measurements were performed and Boolean gating strategy was adapted on the ISHAGE guidelines for CD34^+^ cells. As described before, parameters defined as optimal for the targeting approach were used for this purpose and untransfected cells served as internal control.

**Figure 6:**
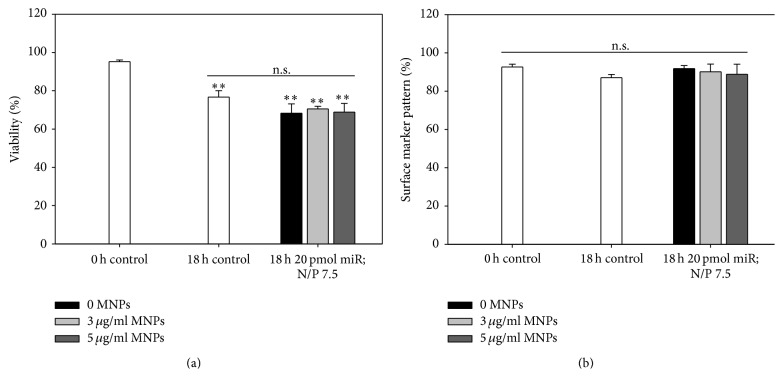
Viability and surface marker pattern after transfection. Cells were transfected with optimized miR/PEI (20 pmol miR; N/P ratio 7.5) and miR/PEI/MNP (20 pmol miR; N/P ratio 7.5; 3 and 5 *μ*g/mL MNPs) complexes. Viability (a) and expression of CD45, CD34, and CD133 (b) were measured 18 h after transfection by flow cytometry. Untransfected cells (0 h and 18 h) were used as control. Values are presented as mean ± SEM; *n* = 3; statistic was performed versus 0 h control (indicated as *∗*) or versus 18 h control; ^*∗*^ *p* ≤ 0.05; ^*∗∗*^ *p* ≤ 0.01; ^*∗∗∗*^ *p* ≤ 0.001.

A significant decrease in the viability of untransfected CD133^+^ cells from 95 ± 1% (0 h control) to 77 ± 3% (18 h control) was observed (Figure 6). This represented cell death after 18 h culturing time most likely resulted from the suboptimal content of DMEM culture medium selected for the establishment of the transfection system. At the same time, no significant difference in viability was determined between control and cells transfected with 20 pmol miR, N/P 7.5, and 0, 3, and 5 *μ*g/mL MNPs (~70% living cells) after 18 h, illustrating that the chosen transfection setup itself did not affect cell viability.

Notably, no significant changes in cell surface marker pattern (CD45, CD34, and CD133) were observed after 18 h culturing time (93 ± 2% 0 h control, 87 ± 2% 18 h control). Moreover, analysis of cells transfected with 20 pmol miR, N/P 7.5, and 0, 3, and 5 *μ*g/mL MNPs (92 ± 2%, 90 ± 4%, and 89 ± 4%) showed no significant difference compared to both control time points, indicating the preservation of stem cell characteristics.

### 3.7. Modified CD133^+^ Stem Cells Retain Their Haematopoietic Differentiation Potential

**Figure 7:**
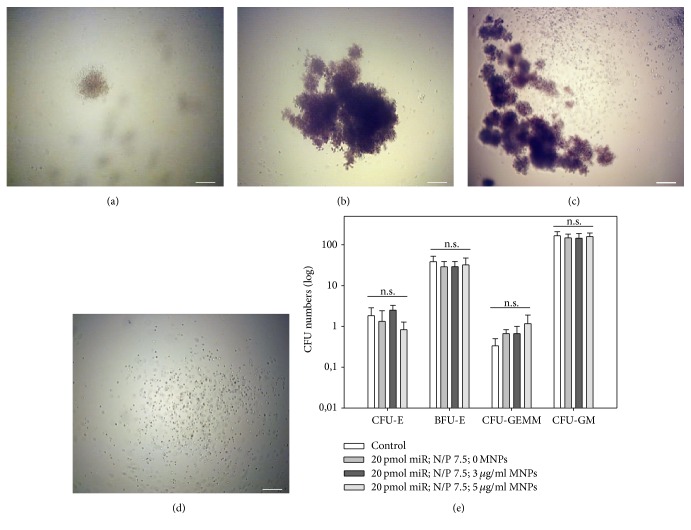
Haematopoietic differentiation capacity of cells after transfection. Cells were transfected with optimized miR/PEI (20 pmol miR; N/P ratio 7.5) and miR/PEI/MNP (20 pmol miR; N/P ratio 7.5; 3 and 5 *μ*g/mL MNPs) complexes and colony-forming unit (CFU) assays were performed. After incubation for 14 d, CFU-erythroid (CFU-E) (a), Burst-forming unit-erythroid (BFU-E) (b), CFU-granulocyte, erythroid, macrophage, and megakaryocyte (CFU-GEMM) (c), and CFU-granulocyte and macrophage (CFU-GM) (d) were counted (e). Presented pictures are illustrating observed cell morphologies. Untransfected cells were used as control. Values are presented as mean ± SEM; *n* = 3; statistic was performed within respective units; ^*∗*^ *p* ≤ 0.05; ^*∗∗*^ *p* ≤ 0.01; ^*∗∗∗*^ *p* ≤ 0.001. Scale bar = 200 *μ*m.

BM derived CD133^+^ cells are known to bear multipotent differentiation potential. In order to address a potential influence of our transfection system on their haematopoietic differentiation capacity, CFU-H assay was utilized. The comparison of untransfected cells (control) and cells modified with optimized miR/PEI and miR/PEI/MNP complexes (20 pmol miR; N/P ratio 7.5; 0, 3, and 5 *μ*L/mL MNPs) showed no significant difference in the amount of formed CFU-granulocyte, erythroid, macrophage, megakaryocyte (CFU-GEMM), CFU-granulocyte, macrophage (CFU-GM), Burst-forming unit-erythroid (BFU-E), and CFU-erythroid (CFU-E) (Figure 7(e)). Moreover, CFUs formed by transfected cells had no morphological abnormalities (Figures 7(a)–7(d)). These results clearly demonstrate that transfection under optimized conditions has no influence on haematopoietic differentiation potential of CD133^+^ cells.

### 3.8. Modified CD133^+^ Cells Retain Cell Viability and Surface Marker Pattern in Supportive Cytokine-Supplemented Culture Medium

**Figure 8:**
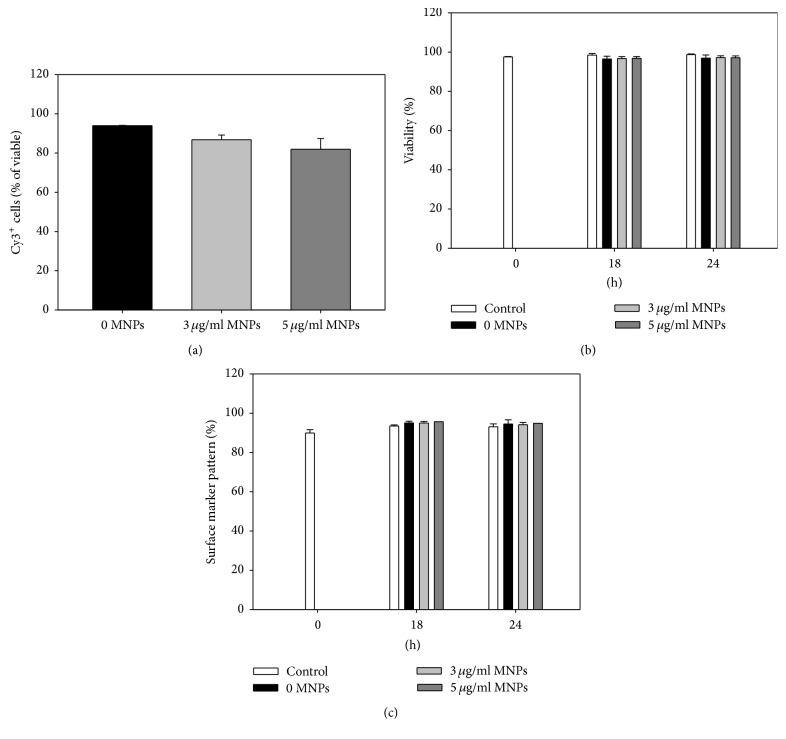
Uptake efficiency, viability, and surface marker pattern after transfection in supportive cytokine-supplemented culture medium. Cells were transfected with optimized miR/PEI (20 pmol miR; N/P ratio 7.5) and miR/PEI/MNP (20 pmol miR; N/P ratio 7.5; 3 and 5 *μ*g/mL MNPs) complexes and cultured in StemSpan H3000 supplemented with StemSpan CC100. Uptake efficiency (a) was measured 18 h after transfection by flow cytometry and viability (b) and expression of CD45, CD34, and CD133^+^ (c) were measured 18 h and 24 h after transfection by flow cytometry. Untransfected cells were used as control. Values are presented as mean ± SEM; *n* = 2.

In the initial transfection setup, 18 h was sufficient for transfection but cell viability was affected when DMEM medium was used for culturing. Therefore, we further have tested a different culture medium suitable for haematopoietic stem cells (HSCs) (StemSpan supplemented with StemSpan CC100) in order to apply the optimized transfection system in a clinically more relevant scenario (Figure 8). We have observed that this more complex medium did not impair the high transfection efficiencies of the system: 87 ± 2% and 82 ± 5% for 20 pmol miR, N/P 7.5 combined with 3 and 5 *μ*g/mL MNPs, respectively, in StemSpan versus 79 ± 9% and 80 ± 8% in DMEM. Yet, in StemSpan, cell viability remained unaffected 18 h and 24 h after transfection compared to 0 h, whereas in DMEM it was reduced by ~25% dead cells after 18 h. Moreover, cultivation of CD133^+^ cells in StemSpan caused no changes in the expression of stem cell surface markers (CD45, CD34, and CD133) after an incubation of 18 h and 24 h of control as well as transfected cells.

## 4. Discussion

Several phases, I and II, clinical studies have shown that CD133^+^ stem cell application for the treatment of various diseases (e.g., liver disease, critical limb ischemia, amyotrophic lateral sclerosis, spinal cord injuries, cerebral palsy, chronic ischemic cardiomyopathy, and leukaemia) is safe and feasible [10, 55–65]. However, despite the large amount of collected clinical data, the benefit seen in trials is inconsistent and small. This underlines the urgent need of further optimization of the cells for an improved clinical outcome.

Therefore, we developed a nonviral multifunctional transfection system which may become a basis to improve the properties of freshly isolated human CD133^+^ stem cells for innovative therapies. This multifunctional system enables efficient miR delivery (~80%) with no significant cytotoxic effects. Moreover, the proposed system ensures magnetic guidance to the site of interest. Simultaneously, we demonstrated that our system has influence neither on stem cell marker expression nor on the haematopoietic differentiation capacity of cells.

The introduction of miRs into stem cells can improve cell survival and engraftment by posttranscriptional gene regulation [39, 40, 66–68]. Furthermore, miR mediated cell modification can be used for programming of CD133^+^ stem cells into specific cell types. Recently, it was shown that miR-146a, miR-150, and miR-451 promote the differentiation of CD133^+^ stem cells into t-lymphoid and erythroid lineages, respectively [69, 70]. This may become valuable in the production of artificial blood for avoiding allogenic blood transfusion after chemotherapy or in the treatment of blood disorders [69]. Yet, in all of these studies, the virus-based delivery of miR into CD133^+^ stem cells was used. However, clinical translation of viruses is still hampered by safety issues including potential immunogenicity and insertional mutagenesis [68].

To reach a safe and efficient way for nonviral miR delivery into CD133^+^ stem cells, we initially tested miR/PEI complex with different compositions. PEI is one of the most efficient reagents for gene delivery and has been used in several clinical trials proving its safety and biocompatibility [71]. Due to the high cationic charge, PEI forms stable complexes with miR which enter the cell through endocytosis [72]. Subsequently, PEI promotes endosomal escape by causing influx of protons and water (proton sponge effect) leading to endosome swelling and disruption to release complexes into the cytoplasm [73]. However, the main drawback of PEI is its potential cytotoxicity after its intracellular accumulation [74]. Keeping in mind this toxicity issue, we selected complexes composed of low miR (20 pmol per 5 × 10^4^ cells) and PEI (N/P ratios 2.5–7.5) amounts for CD133^+^ stem cell engineering, although higher amounts led to improved uptake efficiencies (Figure 2).

In the next step, miR/PEI complexes were combined with MNPs via biotin-streptavidin bonds, which enabled efficient magnetic targeting* in vitro* (Figure 4). In addition, the presence of a magnetic core adds the potential for noninvasive MRI tracking [51]. The application of MNPs is a well investigated, multifunctional tool, which has been used for monitoring, magnetic drug targeting, magnetic fluid hyperthermia, and magnetic labeling and separation of cells [51, 75]. Recently, it was shown that magnetic field based delivery of magnetically labeled CD133^+^ stem cells improved cell retention at the site of interest and enhanced the repair of skeletal muscle and spinal cord injury [76, 77]. However, we have been able to demonstrate that MACS CD133 MicroBeads alone are not sufficient for magnetic targeting of cells* in vitro* (Figure 4). For this reason, we evaluate the influence of utilized MNPs. Therefore, CD133^+^ stem cells were transfected with previously selected miR/PEI complexes complemented with six different MNP amounts (0, 1, 2, 3, 4, and 5 *μ*g/mL) (Figure 3). Our current results showed no significant differences in uptake efficiencies and cytotoxicity of miR/PEI/MNP complexes compared to respective miR/PEI complexes. This result is in correspondence with previous studies, where no differences between magnetic and nonmagnetic complexes were found after transfection of freshly isolated CD105^+^ human mesenchymal stem cells [78]. In contrast, the amount of PEI influenced uptake efficiency: for each MNP concentration, higher NP ratios correlated with higher numbers of Cy3-positive cells. This observation can be easily explained by the widely described fact that unbound PEI is necessary for the nucleic acid delivery [79–81]. Since we have detected no increase in toxicity of complexes formed by 20 pmol miR per 5 × 10^4^ cells, N/P ratio 7.5, and 0–5 *μ*g/mL MNPs as compared to untransfected cells (control), these complexes were considered as optimal for transfection of CD133^+^ cells.

At the same time, however, it should be noted that remarkable variations in viability and transfection efficiency were detected among cells from different donors. This observation corresponds to previous findings of our group, where BM derived hMSCs were used for transfection [46, 82]. Yet, it is not clear which inherent differences from patients lead to the individual variations and further studies should be considered prior to the clinical translation. Moreover, measuring uptake efficiency of Cy3 dye-labeled Pre-miR Negative Control #1 does not allow showing the miR processing within cells. However, previously obtained results by our group should be taken into account: the optimal Cy3-miR transfection conditions resulted in sufficient release and effect of miR once tested with functional miRs and evaluated by qPCR [47, 83]. Therefore, we assume that miR delivery into CD133^+^ cells indicates also an efficient cell transfection.

Moreover, we intended to study the targeting potential of modified CD133^+^ stem cells* in vitro*. For this purpose, a magnet was attached under the culture plate. As a result, significantly higher amounts of cells were located in the area over the magnet versus the area without magnet (Figure 4). In contrast, untransfected cells showed almost the same cell numbers in both areas. This demonstrates that cells transfected with our magnet-bead based system can be efficiently guided to specific locations in the culture plate using a magnetic field. Therefore, a sufficient amount of incorporated iron was defined as 0.155 pg per cell by MPS. However, the guidance is limited to a certain degree (approximately one out of four cells was not targeted) and increased MNP amounts did not lead to improved targeting ratios (2.2 ± 0.08 with 5 *μ*g/mL MNPs). This observation can be explained by hypothesizing that nontargeted CD133^+^ stem cells include the percentage of untransfected cells (~20%) and dead cells. This might also explain lower targeting ratios (between 1.6 and 1.9) after transfecting cells with complexes showing lower uptake rates (~60% using N/P ratio 5). To conclude, performed* in vitro* targeting experiments demonstrate that the localisation of modified cells can be potentially controlled by applying a magnetic field. This potential might become crucial to overcome initial cell washout from the injured tissue and therefore be beneficial to reach better long-term engraftment and functional benefits* in vivo*. However, many factors in internal environment can affect cell retention in both negative and positive ways; therefore, future* in vivo* trials are clearly necessary.

In addition, successfully applied protocol for 3-colour labeling of complex components and delivered nucleic acid might be applied to track the vector fate in tissues after cell transplantation, when progressing to* in vivo* stage.

Importantly, we could demonstrate that our system influences neither the level of stem cell marker expression (CD34 and CD133 based on ISHAGE guidelines) (Figures 6(a) and 8(a)) nor the differentiation into several haematopoietic lineages (Figure 7). Despite this fact, initial culture conditions do affect cell viability. In particular, we discovered a notable decrease (~25%) of CD133^+^ stem cell viability after 18 h cultivation time at 37°C and 5% CO_2_ in DMEM. This observation corresponds with previous studies of our group, where a significant loss of CD133^+^ stem cell viability 72 h after isolation was shown, even under 4°C storage [84]. To date, many efforts have been devoted to the selection of proper supportive medium for HSCs. The combination of SCF and Flt3L with IL-11 or Tpo cytokines was defined as most supportive for HSC survival, proliferation, and maintenance* in vitro* and therefore represents the basic supplement in respective types of medium such as StemSpan [85, 86]. However, very few information is available on suitability of such medium for transfection, in particular when cationic polymer based delivery vectors are applied [87]. Therefore, we have selected simple DMEM medium supplemented with FBS and antibiotics for the establishment of our transfection system. Thereby, we have proven the suitability of our miR/PEI/MNP system for effective miR delivery as well as for magnetic cell targeting. At the same time, the culture conditions were proven to be suboptimal for cell viability and expansion: already after 18 h, viability decreased to ~70–75%. Therefore, we have examined the possibility to efficiently transfect CD133^+^ cells in a more clinically relevant medium supplemented with supporting cytokines. We have observed that in StemSpan H3000 (xeno- and serum-free) supplemented with StemSpan CC100 suitable for HSC culture, transfection worked equally as in DMEM in terms of high efficiency: 80–90% of cells had taken up Cy3-miR. Importantly, cell viability as well as expression of stem cell surface markers remained unaffected after 18 h and 24 h in culture. Taken together, our data prove that PEI/MNP based miR delivery is suitable for gentle and safe CD133^+^ stem cell engineering and for further preclinical investigation.

## 5. Conclusion

In this study, we developed a gentle and safe strategy for efficient modification of human CD133^+^ stem cells by miR. This strategy is based on the nonviral transfection system consisting of biotinylated PEI bound to streptavidin-coated iron oxide MNPs designed in our group. Optimized transfection complexes are suitable to reach high miR uptake rates (~80–90%) without affecting CD133^+^ stem cell characteristics. In addition, using optimally supplemented supportive medium, cell viability remains unaffected after transfection. Furthermore, we demonstrated that modified cells can be magnetically guided to the site of interest* in vitro*. Hence, we expect our magnet-bead based miR delivery system to become an important tool for the engineering of stem cells prior to transplantation which can address certain challenges such as noninvasive cellular* in vivo* monitoring, low cell retention and initial cell death, and directed cell differentiation.

## Supplementary Material

Supplementary Material includes representative figures. They illustrate gating strategies applied in flow cytometry and laser scanning confocal visualization of transfected CD133+ (performed prior to superresolution SIM imaging).

## References

[1] Girlovanu M., Susman S., Soritau O. (2015). Stem cells—biological update and cell therapy progress. *Clujul Medical*.

[2] Meregalli M., Farini A., Belicchi M., Torrente Y. (2010). CD133^+^ cells isolated from various sources and their role in future clinical perspectives. *Expert Opinion on Biological Therapy*.

[3] Lee S., Yoon Y.-S. (2013). Revisiting cardiovascular regeneration with bone marrow-derived angiogenic and vasculogenic cells. *British Journal of Pharmacology*.

[4] Beksac M., Preffer F. (2012). Is it time to revisit our current hematopoietic progenitor cell quantification methods in the clinic. *Bone Marrow Transplantation*.

[5] Bongiovanni D., Bassetti B., Gambini E. (2014). The CD133^+^ cell as advanced medicinal product for myocardial and limb ischemia. *Stem Cells and Development*.

[6] Wang X., Zhang J., Zhang F. (2015). The clinical status of stem cell therapy for ischemic cardiomyopathy. *Stem Cells International*.

[7] Ma N., Ladilov Y., Moebius J. M. (2006). Intramyocardial delivery of human CD133+ cells in a SCID mouse cryoinjury model: bone marrow vs. cord blood-derived cells. *Cardiovascular Research*.

[8] Rafii S., Lyden D. (2003). Therapeutic stem and progenitor cell transplantation for organ vascularization and regeneration. *Nature Medicine*.

[9] Bartunek J., Vanderheyden M., Vandekerckhove B. (2005). Intracoronary injection of CD133-positive enriched bone marrow progenitor cells promotes cardiac recovery after recent myocardial infarction: feasibility and safety. *Circulation*.

[10] Stamm C., Kleine H.-D., Choi Y.-H. (2007). Intramyocardial delivery of CD133^+^ bone marrow cells and coronary artery bypass grafting for chronic ischemic heart disease: safety and efficacy studies. *The Journal of Thoracic and Cardiovascular Surgery*.

[11] Manginas A., Goussetis E., Koutelou M. (2007). Pilot study to evaluate the safety and feasibility of intracoronary CD133^+^ and CD133^−^ CD34^+^ cell therapy in patients with nonviable anterior myocardial infarction. *Catheterization and Cardiovascular Interventions*.

[12] Colombo A., Castellani M., Piccaluga E. (2011). Myocardial blood flow and infarct size after CD133+ cell injection in large myocardial infarction with good recanalization and poor reperfusion: results from a randomized controlled trial. *Journal of Cardiovascular Medicine*.

[13] Yerebakan C., Kaminski A., Westphal B. (2011). Impact of preoperative left ventricular function and time from infarction on the long-term benefits after intramyocardial CD133^+^ bone marrow stem cell transplant. *The Journal of Thoracic and Cardiovascular Surgery*.

[14] Wang D., Gao G. (2014). State-of-the-art human gene therapy: part I. Gene delivery technologies. *Discovery Medicine*.

[15] Sart S., Ma T., Li Y. (2014). Preconditioning stem cells for in vivo delivery. *BioResearch Open Access*.

[16] Liu J., Narsinh K. H., Lan F. (2012). Early stem cell engraftment predicts late cardiac functional recovery: preclinical insights from molecular imaging. *Circulation: Cardiovascular Imaging*.

[17] Terrovitis J. V., Smith R. R., Marbán E. (2010). Assessment and optimization of cell engraftment after transplantation into the heart. *Circulation Research*.

[18] Lang C., Lehner S., Todica A. (2014). In-vivo comparison of the acute retention of stem cell derivatives and fibroblasts after intramyocardial transplantation in the mouse model. *European Journal of Nuclear Medicine and Molecular Imaging*.

[19] Rosado-de-Castro P. H., Schmidt F. R., Battistella V. (2013). Biodistribution of bone marrow mononuclear cells after intra-arterial or intravenous transplantation in subacute stroke patients. *Regenerative Medicine*.

[20] Kang W. J., Kang H., Kim H., Chung J., Lee M. C., Lee D. S. (2006). Tissue distribution of ^18^F-FDG-labeled peripheral hematopoietic stem cells after intracoronary administration in patients with myocardial infarction. *Journal of Nuclear Medicine*.

[21] Goussetis E., Manginas A., Koutelou M. (2006). Intracoronary infusion of CD133^+^ and CD133^−^CD34^+^ selected autologous bone marrow progenitor cells in patients with chronic ischemic cardiomyopathy: cell isolation, adherence to the infarcted area, and body distribution. *Stem Cells*.

[22] Blocklet D., Toungouz M., Berkenboom G. (2006). Myocardial homing of nonmobilized peripheral-blood CD34^+^ cells after intracoronary injection. *Stem Cells*.

[23] Penicka M., Lang O., Widimsky P. (2007). One-day kinetics of myocardial engraftment after intracoronary injection of bone marrow mononuclear cells in patients with acute and chronic myocardial infarction. *Heart*.

[24] Caveliers V., de Keulenaer G., Everaert H. (2007). In vivo visualization of 111In labeled CD133^+^ peripheral blood stem cells after intracoronary administration in patients with chronic ischemic heart disease. *The Quarterly Journal of Nuclear Medicine and Molecular Imaging*.

[25] Schächinger V., Aicher A., Döbert N. (2008). Pilot trial on determinants of progenitor cell recruitment to the infarcted human myocardium. *Circulation*.

[26] Dedobbeleer C., Blocklet D., Toungouz M. (2009). Myocardial homing and coronary endothelial function after autologous blood CD34+ progenitor cells intracoronary injection in the chronic phase of myocardial infarction. *Journal of Cardiovascular Pharmacology*.

[27] Musialek P., Tekieli L., Kostkiewicz M. (2011). Randomized transcoronary delivery of CD34^+^ cells with perfusion versus stop-flow method in patients with recent myocardial infarction: early cardiac retention of ^99^mTc-labeled cells activity. *Journal of Nuclear Cardiology*.

[28] Kyrtatos P. G., Lehtolainen P., Junemann-Ramirez M. (2009). Magnetic tagging increases delivery of circulating progenitors in vascular injury. *JACC: Cardiovascular Interventions*.

[29] Vandergriff A. C., Hensley T. M., Henry E. T. (2014). Magnetic targeting of cardiosphere-derived stem cells with ferumoxytol nanoparticles for treating rats with myocardial infarction. *Biomaterials*.

[30] Huang Z., Shen Y., Sun A. (2013). Magnetic targeting enhances retrograde cell retention in a rat model of myocardial infarction. *Stem Cell Research & Therapy*.

[31] Yanai A., Häfeli U. O., Metcalfe A. L. (2012). Focused magnetic stem cell targeting to the retina using superparamagnetic iron oxide nanoparticles. *Cell Transplantation*.

[32] Arbab A. S., Jordan E. K., Wilson L. B., Yocum G. T., Lewis B. K., Frank J. A. (2004). In Vivo trafficking and targeted delivery of magnetically labeled stem cells. *Human Gene Therapy*.

[33] Cores J., Caranasos T., Cheng K. (2015). Magnetically targeted stem cell delivery for regenerative medicine. *Journal of Functional Biomaterials*.

[34] Mejías R., Gutiérrez L., Salas G. (2013). Long term biotransformation and toxicity of dimercaptosuccinic acid-coated magnetic nanoparticles support their use in biomedical applications. *Journal of Controlled Release*.

[35] Zheng B., von See M. P., Yu E. (2016). Quantitative magnetic particle imaging monitors the transplantation, biodistribution, and clearance of stem cells in vivo. *Theranostics*.

[36] Noort W. A., Feye D., van Den Akker F. (2010). Mesenchymal stromal cells to treat cardiovascular disease: strategies to improve survival and therapeutic results. *Panminerva Medica*.

[37] Don C. W., Murry C. E. (2013). Improving survival and efficacy of pluripotent stem cell-derived cardiac grafts. *Journal of Cellular and Molecular Medicine*.

[38] Robey T. E., Saiget M. K., Reinecke H., Murry C. E. (2008). Systems approaches to preventing transplanted cell death in cardiac repair. *Journal of Molecular and Cellular Cardiology*.

[39] Jakob P., Landmesser U. (2012). Role of microRNAs in stem/progenitor cells and cardiovascular repair. *Cardiovascular Research*.

[40] Sen C. K. (2011). MicroRNAs as new maestro conducting the expanding symphony orchestra of regenerative and reparative medicine. *Physiological Genomics*.

[41] Papapetrou E. P., Zoumbos N. C., Athanassiadou A. (2005). Genetic modification of hematopoietic stem cells with nonviral systems: past progress and future prospects. *Gene Therapy*.

[42] Chira S., Jackson C. S., Oprea I. (2015). Progresses towards safe and efficient gene therapy vectors. *Oncotarget*.

[43] Douglas K. L. (2008). Toward development of artificial viruses for gene therapy: a comparative evaluation of viral and non-viral transfection. *Biotechnology Progress*.

[44] Höbel S., Aigner A. (2013). Polyethylenimines for siRNA and miRNA delivery in vivo. *Wiley Interdisciplinary Reviews: Nanomedicine and Nanobiotechnology*.

[45] Cubillos-Ruiz J. R., Sempere L. F., Conejo-Garcia J. R. (2012). Good things come in small packages: therapeutic anti-tumor immunity induced by microrna nanoparticles. *OncoImmunology*.

[46] Delyagina E., Schade A., Scharfenberg D. (2014). Improved transfection in human mesenchymal stem cells: effective intracellular release of pDNA by magnetic polyplexes. *Nanomedicine*.

[47] Schade A., Delyagina E., Scharfenberg D. (2013). Innovative strategy for microRNA delivery in human mesenchymal stem cells via magnetic nanoparticles. *International Journal of Molecular Sciences*.

[48] Sutherland D. R., Anderson L., Keeney M., Nayar R., Chin-Yee I. (1996). The ISHAGE guidelines for CD34^+^ cell determination by flow cytometry. *Journal of Hematotherapy and Stem Cell Research*.

[49] Li W., Ma N., Ong L.-L. (2008). Enhanced thoracic gene delivery by magnetic nanobead-mediated vector. *The Journal of Gene Medicine*.

[50] Kircheis R., Wightman L., Schreiber A. (2001). Polyethylenimine/DNA complexes shielded by transferrin target gene expression to tumors after systemic application. *Gene Therapy*.

[51] Almstätter I., Mykhaylyk O., Settles M. (2015). Characterization of magnetic viral complexes for targeted delivery in oncology. *Theranostics*.

[52] Scharlach C., Kratz H., Wiekhorst F. (2015). Synthesis of acid-stabilized iron oxide nanoparticles and comparison for targeting atherosclerotic plaques: evaluation by MRI, quantitative MPS, and TEM alternative to ambiguous Prussian blue iron staining. *Nanomedicine: Nanotechnology, Biology, and Medicine*.

[53] Harms C., Datwyler A. L., Wiekhorst F. (2013). Certain types of iron oxide nanoparticles are not suited to passively target inflammatory cells that infiltrate the brain in response to stroke. *Journal of Cerebral Blood Flow and Metabolism*.

[54] Poller W. C., Löwa N., Wiekhorst F. (2016). Magnetic particle spectroscopy reveals dynamic changes in the magnetic behavior of very small superparamagnetic iron oxide nanoparticles during cellular uptake and enables determination of cell-labeling efficacy. *Journal of Biomedical Nanotechnology*.

[55] King A., Barton D., Beard H. A. (2015). REpeated AutoLogous Infusions of STem cells in Cirrhosis (REALISTIC): a multicentre, phase II, open-label, randomised controlled trial of repeated autologous infusions of granulocyte colony-stimulating factor (GCSF) mobilised CD133+ bone marrow stem cells in patients with cirrhosis. A study protocol for a randomised controlled trial. *BMJ Open*.

[56] Moore J. K., Stutchfield B. M., Forbes S. J. (2014). Systematic review: the effects of autologous stem cell therapy for patients with liver disease. *Alimentary Pharmacology & Therapeutics*.

[57] Martínez H. R., Molina-Lopez J. F., González-Garza M. T. (2012). Stem cell transplantation in amyotrophic lateral sclerosis patients: methodological approach, safety, and feasibility. *Cell Transplantation*.

[58] Jimenez-Quevedo P., Gonzalez-Ferrer J. J., Sabate M. (2014). Selected CD133^+^ progenitor cells to promote angiogenesis in patients with refractory angina: final results of the PROGENITOR randomized trial. *Circulation Research*.

[59] Raval A. N., Schmuck E. G., Tefera G. (2014). Bilateral administration of autologous CD133^+^ cells in ambulatory patients with refractory critical limb ischemia: lessons learned from a pilot randomized, double-blind, placebo-controlled trial. *Cytotherapy*.

[60] Andreone P., Catani L., Margini C. (2016). Reinfusion of highly purified CD133^+^ bone marrow-derived stem/progenitor cells in patients with end-stage liver disease: a phase I clinical trial. *Digestive and Liver Disease*.

[61] Arici V., Perotti C., Fabrizio C. (2015). Autologous immuno magnetically selected CD133^+^ stem cells in the treatment of no-option critical limb ischemia: clinical and contrast enhanced ultrasound assessed results in eight patients. *Journal of Translational Medicine*.

[62] Zali A., Arab L., Ashrafi F. (2015). Intrathecal injection of CD133-positive enriched bone marrow progenitor cells in children with cerebral palsy: feasibility and safety. *Cytotherapy*.

[63] Al-Zoubi A., Jafar E., Jamous M. (2014). Transplantation of purified autologous leukapheresis-derived CD34^+^ and CD133^+^ stem cells for patients with chronic spinal cord injuries: long-term evaluation of safety and efficacy. *Cell Transplantation*.

[64] Isidori A., Motta M. R., Tani M. (2007). Positive selection and transplantation of autologous highly purified CD133^+^ stem cells in resistant/relapsed chronic lymphocytic leukemia patients results in rapid hematopoietic reconstitution without an adequate leukemic cell purging. *Biology of Blood and Marrow Transplantation*.

[65] Nasseri B. A., Ebell W., Dandel M. (2014). Autologous CD133^+^ bone marrow cells and bypass grafting for regeneration of ischaemic myocardium: the Cardio133 trial. *European Heart Journal*.

[66] Urbich C., Kuehbacher A., Dimmeler S. (2008). Role of microRNAs in vascular diseases, inflammation, and angiogenesis. *Cardiovascular Research*.

[67] Ong S.-M., Biswas S. K., Wong S.-C. (2015). MicroRNA-mediated immune modulation as a therapeutic strategy in host-implant integration. *Advanced Drug Delivery Reviews*.

[68] Peng B., Chen Y., Leong K. W. (2015). MicroRNA delivery for regenerative medicine. *Advanced Drug Delivery Reviews*.

[69] Kouhkan F., Hafizi M., Mobarra N. (2014). miRNAs: a new method for erythroid differentiation of hematopoietic stem cells without the presence of growth factors. *Applied Biochemistry and Biotechnology*.

[70] Fallah P., Arefian E., Naderi M. (2013). MiR-146a and miR-150 promote the differentiation of CD133^+^ cells into T-lymphoid lineage. *Molecular Biology Reports*.

[71] Neuberg P., Kichler A. (2014). Recent developments in nucleic acid delivery with polyethylenimines. *Advances in Genetics*.

[72] Midoux P., Breuzard G., Gomez J. P., Pichon C. (2008). Polymer-based gene delivery: a current review on the uptake and intracellular trafficking of polyplexes. *Current Gene Therapy*.

[73] Zhang Y., Wang Z., Gemeinhart R. A. (2013). Progress in microRNA delivery. *Journal of Controlled Release*.

[74] Clamme J.-P., Krishnamoorthy G., Mély Y. (2003). Intracellular dynamics of the gene delivery vehicle polyethylenimine during transfection: investigation by two-photon fluorescence correlation spectroscopy. *Biochimica et Biophysica Acta—Biomembranes*.

[75] Tombácz E., Turcu R., Socoliuc V., Vékás L. (2015). Magnetic iron oxide nanoparticles: recent trends in design and synthesis of magnetoresponsive nanosystems. *Biochemical and Biophysical Research Communications*.

[76] Fujioka Y., Tanaka N., Nakanishi K. (2012). Magnetic field-based delivery of human CD133^+^ cells promotes functional recovery after rat spinal cord injury. *Spine*.

[77] Ohkawa S., Kamei N., Kamei G. (2013). Magnetic targeting of human peripheral blood CD133^+^ Cells for skeletal muscle regeneration. *Tissue Engineering—Part C: Methods*.

[78] Schade A., Müller P., Delyagina E. (2014). Magnetic nanoparticle based nonviral MicroRNA delivery into freshly isolated CD105^+^ hMSCs. *Stem Cells International*.

[79] Ma Y., Zhang Z., Wang X., Xia W., Gu H. (2011). Insights into the mechanism of magnetofection using MNPs-PEI/pDNA/free PEI magnetofectins. *International Journal of Pharmaceutics*.

[80] Hanzlíková M., Ruponen M., Galli E. (2011). Mechanisms of polyethylenimine-mediated DNA delivery: free carrier helps to overcome the barrier of cell-surface glycosaminoglycans. *The Journal of Gene Medicine*.

[81] Yue Y., Jin F., Deng R. (2011). Revisit complexation between DNA and polyethylenimine—effect of length of free polycationic chains on gene transfection. *Journal of Controlled Release*.

[82] Wang W., Li W., Ou L. (2011). Polyethylenimine-mediated gene delivery into human bone marrow mesenchymal stem cells from patients. *Journal of Cellular and Molecular Medicine*.

[83] Voronina N., Lemcke H., Wiekhorst F. (2016). Non-viral magnetic engineering of endothelial cells with microRNA and plasmid-DNA-an optimized targeting approach. *Nanomedicine: Nanotechnology, Biology and Medicine*.

[84] Lux C. A., Mark P., Klopsch C. (2015). Impact of short-term liquid storage on human CD133^+^ stem cells. *Cell Transplantation*.

[85] Walasek M. A., van Os R., de Haan G. (2012). Hematopoietic stem cell expansion: challenges and opportunities. *Annals of the New York Academy of Sciences*.

[86] Sauvageau G., Iscove N. N., Humphries R. K. (2004). In vitro and in vivo expansion of hematopoietic stem cells. *Oncogene*.

[87] Yan M., Wen J., Liang M., Lu Y., Kamata M., Chen I. S. Y. (2015). Modulation of gene expression by polymer nanocapsule delivery of DNA cassettes encoding small RNAs. *PLoS ONE*.

