# Supplementary material for: Magnet-Bead Based MicroRNA Delivery System to Modify CD133+ Stem Cells
Source: Stem Cells Int. 2016 Oct 4;2016:7152761. doi: 10.1155/2016/7152761 (PMC5067480; doi:10.1155/2016/7152761)
Supplement: Supplementary file 1 — Supplementary Material includes representative figures. They illustrate gating strategies applied in flow cytometry and laser scanning confocal visualization of transfected CD133+ (performed prior to superresolution SIM imaging). [file 7152761.f1.docx]

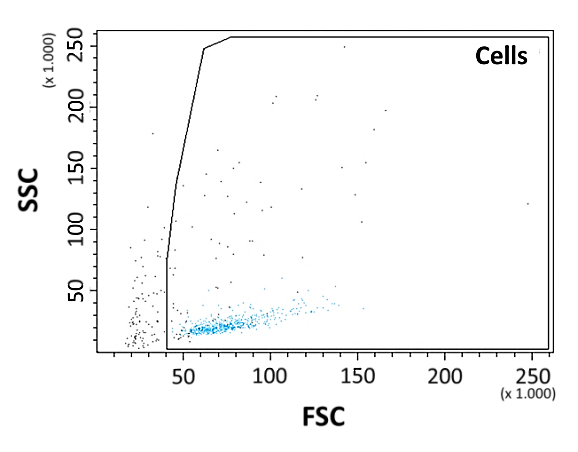

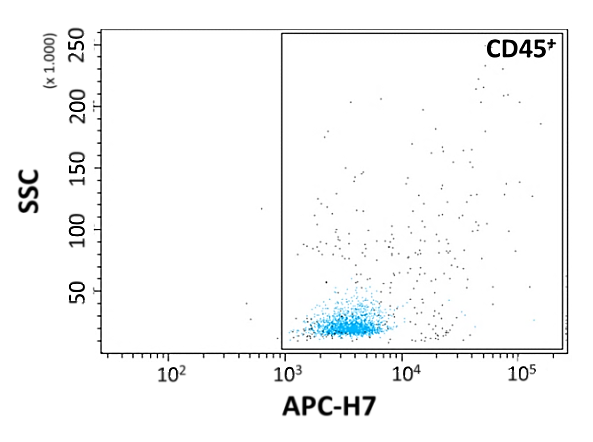

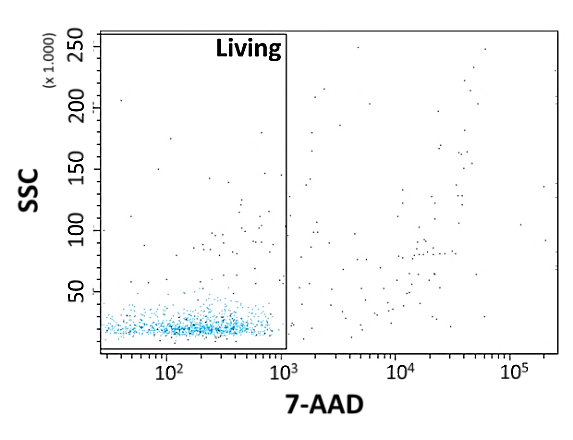

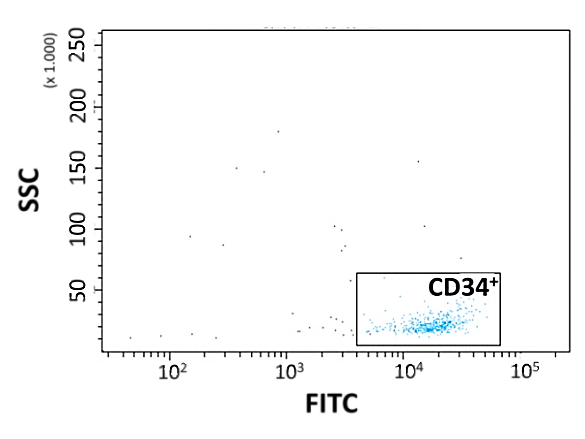

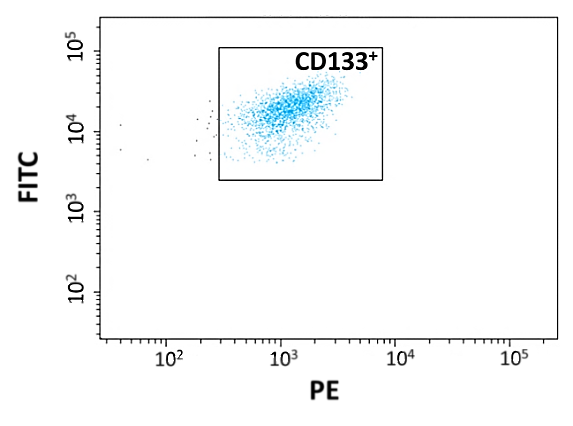

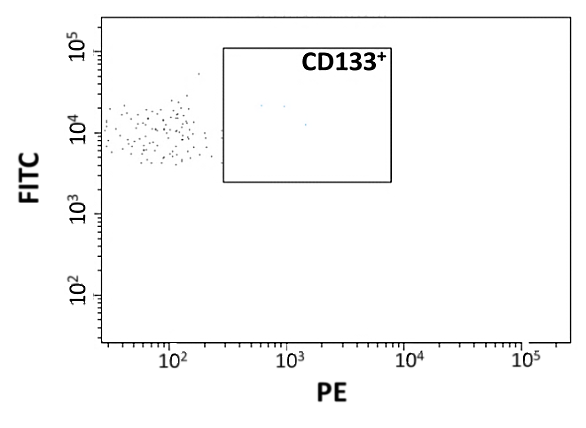


**B**

**A**

**D**

**C**

**F**

**E**

**Figure S1: Boolean gating strategy for flow cytometry measurements of cell viability and surface marker pattern.** (A) Debris was excluded from the intact cell population; (B) CD45^+^ cells (APC-H7 pos.); (C) Viable cells (7-AAD neg.) out of CD45^+^ population; (D) CD34^+^ cells (FITC pos.) out of viable CD45^+^ population; (E) CD133^+^ cells (PE pos.) out of viable CD45^+^/CD34^+^ population; (F) PE isotype control. Blue: viable CD45^+^/CD34^+^/CD133^+^ cells


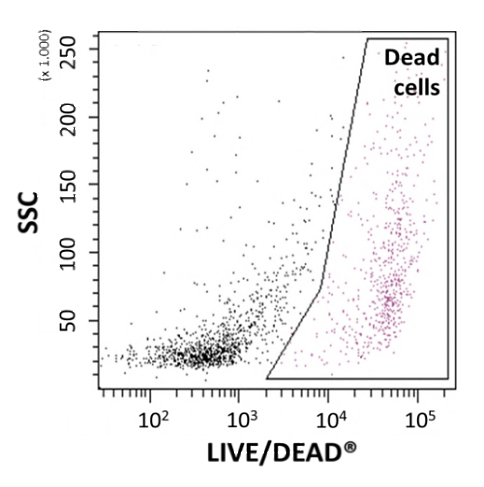

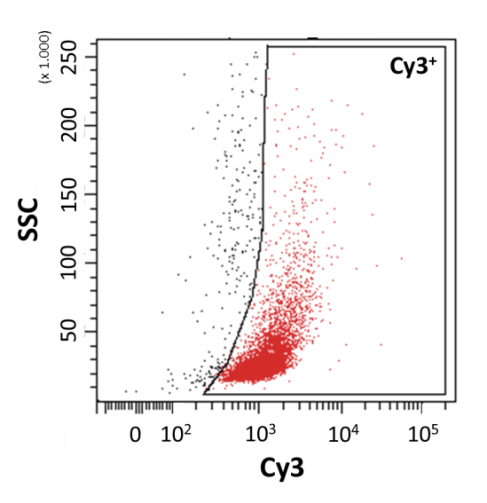

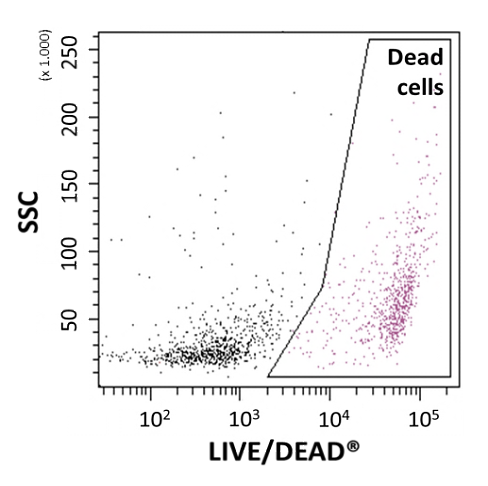

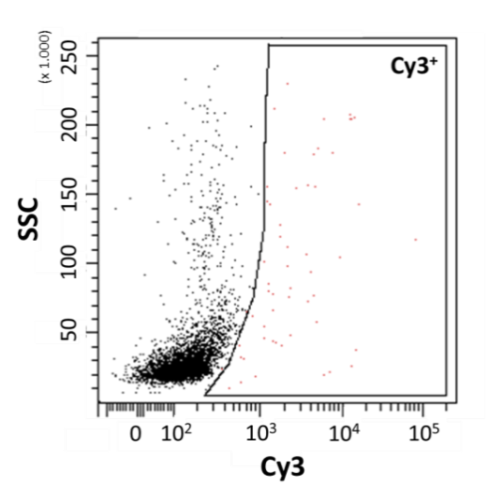

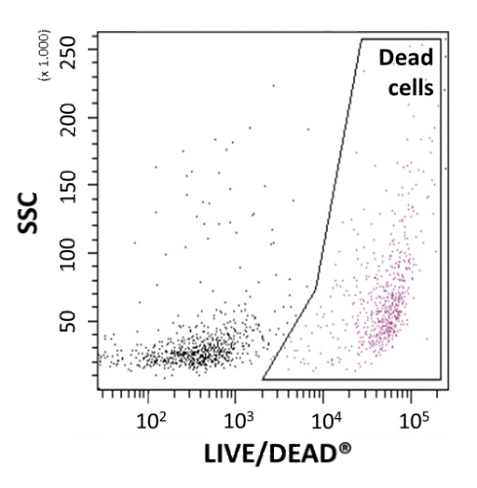

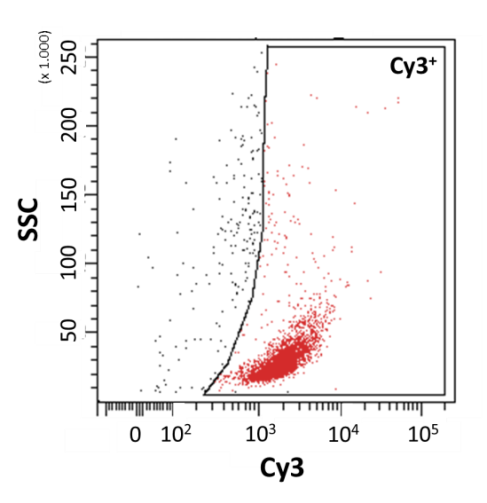


**F**

**E**

**D**

**C**

**B**

**A**

**Figure S2: Gating strategy for flow cytometry measurements of uptake efficiency and cytotoxicity of transfection complexes.** (A-C) Uptake efficiency analyses of miR/PEI (B) and miR/PEI/MNP (C) complexes were based on Cy3-labeling. Unlabeled miR/PEI complexes (A) were used as control. (D - F) Cytotoxic effects of miR/PEI (E) and miR/PEI/MNP (F) complexes were based on labeling dead cells with LIVE/DEAD® Fixable Near-IR Dead Cell staining solution. Untransfected cells (D) were used as control. Red: Cy3^+^ cells; Purple: dead cells.


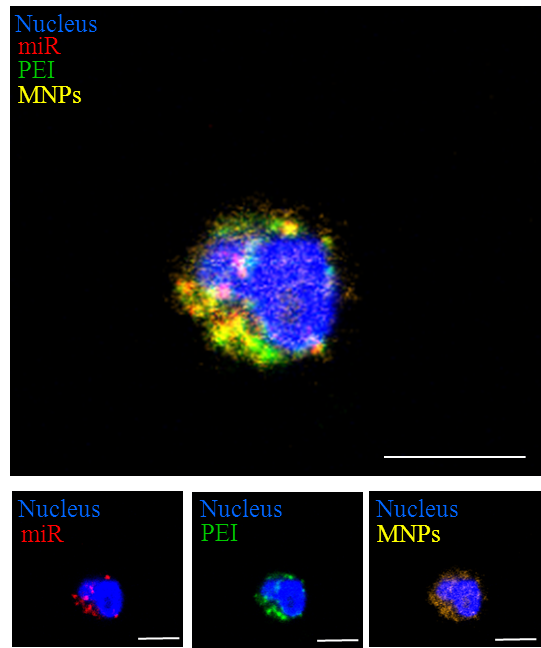


**Figure S3: Intracellular visualization of transfection complexes.** Cells were transfected with optimized fluorescently labeled miR/PEI/MNP (20 pmol miR; N/P ratio 7.5; 3 µg/ml MNPs) complexes. miR staining was performed with Cy5™ dye (red). PEI was labeled Oregon Green^®^ 488 (green). MNPs were stained with Atto 565 (yellow). Nuclei were counterstained with DAPI (blue). Representative images were taken 18h after transfection using laser scanning confocal microscopy. Scale bar = 5 µm.
